# Monthly Summary

**Published:** 1891-10

**Authors:** 


### Monthly Summary.

INSANITY FOLLOWING EXTRACTION OF NINETEEN TEETH.—Mrs. Ella Targett, a woman of large frame was taken to the Bellevue Hospital for the Insane. The husband said he believed his wife's insanity had been brought on by a sitting at the dentist's at which she had nineteen teeth drawn under laughing gas.

"For the past six months my wife has been weak and nervous. She had been in the country for a time, and upon her return complained that she had suffered dreadfully from neuralgia of the face. She thought that she would be better if she had her teeth pulled and begged me to give her money for this purpose. I did so, and she started out with her aunt a week ago last Thursday. She left the house early in the afternoon, but did not return until about 9 o'clock at night. She seemed utterly broken down when she reached the house and was in a fever. For an hour I could not get a word from her as to the experience she had undergone. The aunt told me, however, that my wife lost two quarts of blood, and finally my wife herself said that, although she had taken gas, she could feel each tooth as it was pulled. The aunt declared that she had first asked the dentist if it would be perfectly safe to administer the anæsthetic, and he answered her that it would. A physician who had attended my wife before was called in. He said she ought not to have taken gas. She grew steadily worse, and the insanity under which she now suffers has rapidly developed. She has never been out of her mind before. We have been married eleven years."

Dr. Stuart Douglas, under whose care the patient is at Bellevue said yesterday that her condition is extremely serious. "She screeches in horror at the blood and bloody visions pictured by her disordered mind," he said "and writhes and twists with furious strength in the hands of those who seek to restrain her. The suicidal mania is strongly developed."

When asked if the shock from tooth pulling might precipitate insanity in a person with some leaning that way, Dr. Douglas said it was possible. He would hazard no opinion in Mrs. Targett's case, however, but said that he knew of nothing for which the dentist was reprehensible.—*N. Y. Correspondence Phila. Times, July 19.*

---

NO MORE ARTIFICAL TEETH.—Old age is robbed of half its terrors and much of its deformity by the brilliant discovery of a Moscow dentist Dr. Zuamensko, who, according to a possibly over-sanguine Russian contemparary, has delighted the civilized world by his skill in making teeth grow in toothless gums. After experimenting on dogs, he tried the effects of his method in human beings, and the success was complete. The teeth are made of gutta-percha, porcelain, or metal, as may be desired. The root of the false tooth has some holes bored in it. Holes are now bored into the jaw, and into the hole the false tooth is stuck as is a nail in wood.

In a short time a tender growth starts up the cavity of the false tooth, and this growth hardening, the tooth becomes fixed in position. These new teeth can, according to the inventor, be placed in the alveolus of a natural tooth, and thus, when a diseased tooth is pulled out a metal or porcelain substitute can be inserted in its place, without incurring any risk cf transferring disease, as happened in Hunter's days, when the apparently sound teeth of poor persons when transplanted, not infrequently conveyed disease. There are several minor inconsistences in this statement, but it would be ungracious to look such a noble gift in the mouth, especially as, according to dentists of authority, our race is destined eventually to become edentulous.—*Medical Press.*

---

TO STERILIZE INSTRUMENTS WITHOUT DULLING THEM.—(V. Bergmann's method).—To render instruments perfectly aseptic, and to preserve the cutting edges from oxidation, they are boiled for five minutes in a 1 per cent. solution of bicarbonate of soda. They can remain in this solution indefinitely, without rusting or dulling the cutting edge. When required for operation they are taken out, dried with a sterilized piece of gauze, and handed to the operator. Whenever, in the course of operation, they come in contact with anything not aseptic, all that is required to resterilize them is to dip them a few seconds into the boiling solution of sodium bicarbonate.—*Miller, in Med. and Surg. Review.*

---

DERMATOL.—By this name Dr. R. Heintz, of Breslau, designates a new chemical preparation, the basic gallate of bismuth, which he recommends as an antiseptic with varied therapeutic uses, that may be used advantageously in the place of iodoform. Dermatol is a fine, absolutely in-odorous powder, of saffron-yellow color, non-hygroscopic, and unaffected by light and air. As it is insoluble in ordinary vehicles, it can only be employed in powder. Besides its strong antiseptic properties, it has a stimulating and astringent action which makes it valuable in the treatment of wounds and ulcers, in that it hastens cicatrization. On account of its insolubility, poisoning by it is impossible; it does not produce even a local irritation. According to Dr. Heintz, dermatol may be substituted for iodoform in all cases where the latter is indicated. It may also be used internally, in daily doses of 2 grammes (30 grains), in diseases of the digestive tract, especially in the profuse diarrhœa which is present in catarrhal and ulcerous affections of the intestines.—*Revue Medico Pharmaceutique*, June 30, 1891, p. 111.

---

ASEPTINIC ACID.—This acid (*Wiener med. Press*), it appears, was discovered in 1885, by Busse; it is limpid, miscible with water, has scarcely any odor, has an alkaline taste, and is not toxic; in contact with blood or pus, it gives off oxygen, which explains its antiseptic action. Linde has used it in the treatment of diphtheria, tuberculous abscesses, suppurative wounds, burns, phlegmons, etc. Its antiseptic action is superior, he states, to that of corrosive sublimate or iodoform. A 5-per-cent. solution is used for disinfecting instruments and the hands. For dressing wounds a 5- or 10-per cent. solution on gauze may be used.

Aseptinic acid is an excellent hæmostatic; the effect is almost immediate when the pure acid or a 50-per-cent. solution is used; it is efficient, however, if more dilute solutions are employed.—*Revue Medico-Pharmaceutique*, June 30, 1891, p. 111.

---

WHEN THE BRAIN IS IDLE it degenerates as the muscles degenerate in consequence of disuse. The least vigorous and active brains are to be found in the two extremes in the social scale, the very wealthy and the very poor; while the middle classes furnish examples of the highest brain development.

